# First reported case of Simpson–Golabi–Behmel syndrome in a female fetus diagnosed prenatally with chromosomal microarray

**DOI:** 10.1002/ccr3.902

**Published:** 2017-03-17

**Authors:** Heidi Kristine Støve, Naja Becher, Vibike Gjørup, Mette Ramsing, Ida Vogel, Else Marie Vestergaard

**Affiliations:** ^1^ Department of Clinical GeneticsAarhus University HospitalAarhusDenmark; ^2^ Department of Gynecology and ObstetricsAarhus University HospitalAarhusDenmark; ^3^ Department of PathologyAarhus University HospitalAarhusDenmark

**Keywords:** Chromosomal microarray, prenatal diagnosis, Simpson–Golabi–Behmel syndrome, X‐linked

## Abstract

Simpson–Golabi–Behmel syndrome (SGBS) is a rare X‐linked syndrome. Female carriers may have mild manifestations. Macrosomia, polyhydramnios, and kidney and urinary tract anomalies are common findings in male fetuses. We present the first case of a severely affected female fetus with stigmata of SGBS and a deletion involving the GPC3 gene.

## Introduction

Simpson–Golabi–Behmel syndrome (SGBS, OMIM #312870) is a rare X‐linked disorder characterized by pre and postnatal overgrowth, craniofacial dysmorphism, and a broad spectrum of skeletal and visceral anomalies 1. Variable findings associated with the disease include supernumerary nipples, hernias, congenital heart defects, genitourinary defects, mild to severe intellectual disability, vertebral and rib abnormalities, and postaxial polydactyly. Affected individuals are at increased risk of developing embryonal tumors 2.

The association between mutations in the glypican‐3 gene (*GPC3*) localized to chromosome Xq26.2 and SGBS was first identified by Pilia et al. in 1996 3. *GPC3* is considered to play a role in regulation of growth factor activity and is highly expressed in mesodermal embryonic tissues that are prone to overgrowth in SGBS 3, 4. SGBS is believed to be caused by a nonfunctional GPC3 protein 5.

Carrier females may have mild symptoms, but rarely exhibit distinct clinical features and no prenatal cases have been reported. We hereby report on the prenatal findings in a severely affected female fetus with a 91 kb deletion involving exon 2 of the *GPC3* gene detected by chromosomal microarray (CMA).

## Clinical Report

**Figure 1:**
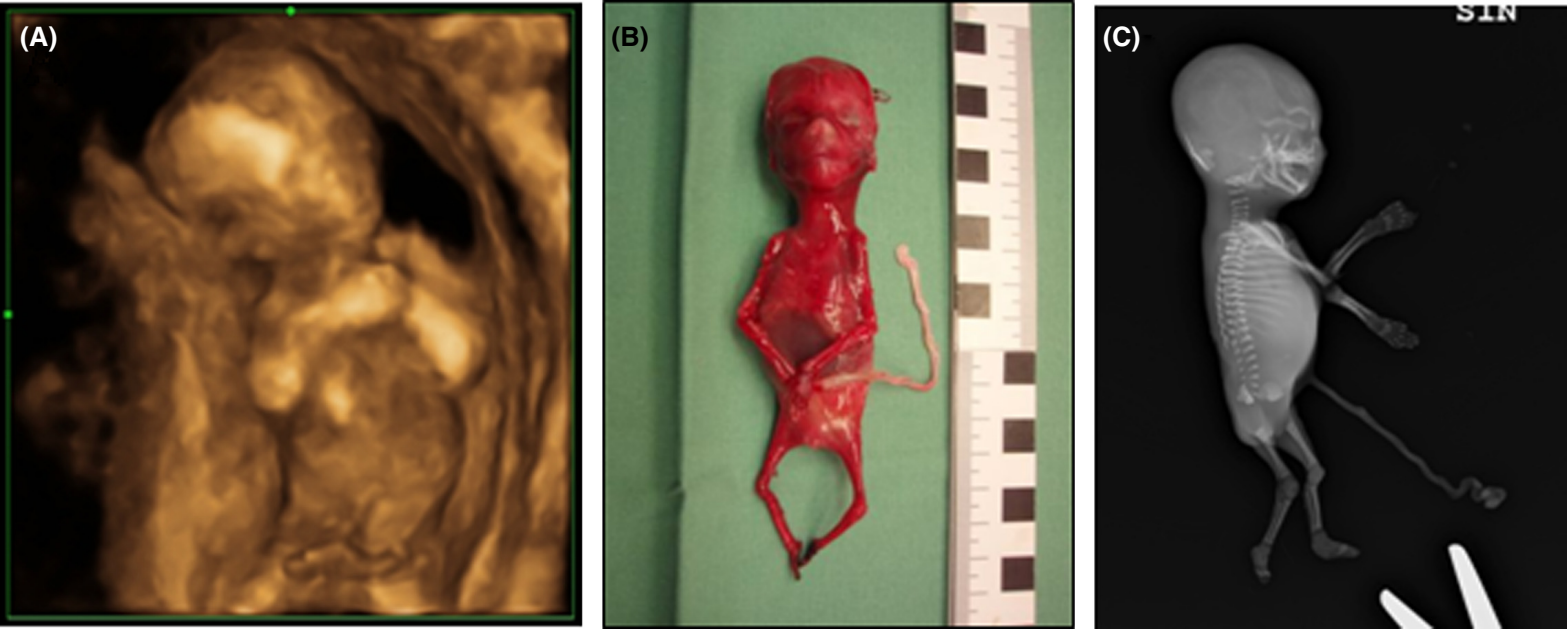
Prenatal appearance at 13 and 15 weeks gestation. (A) Ultrasound image showing the fetus with joint contractures of the upper limbs. (B) Abortus (15 weeks and 2 days) with severe hypoplasia of skeletal muscles, joint contractures, and craniofacial dysmorphism. (C) Radiograph of the fetus at 15 weeks and 2 days gestation.

A 31‐year‐old Caucasian woman was referred for routine first trimester screening at 12 + 5 weeks of gestation. The ultrasound examination revealed a fetus affected with joint contractures with the upper limbs positioned in front of the chest (Fig. 1A) and the lower limbs (not visible in the figure) in a fixed lotus‐like position. Only sparse fetal movements were detected. The nuchal translucency was 2.0 mm. A chorionic villus sampling (CVS) was performed to examine fetal chromosomes. At a repeated ultrasound examination at 14 weeks and 2 days gestation, bilateral polydactyly of the feet was suspected. No heart defects were found.

Rapid test for the common trisomies 21, 13, and 18, and sex chromosome aneuploidies on DNA from CVS, and a standard chromosome analysis on a CVS confirmed a normal female karyotype (46,XX).

Chromosomal microarray (Agilent Technologies Inc., Santa Clara, CA, 180K) was performed because of major structural abnormalities in the fetus and revealed a 91 kb microdeletion at chromosome Xq26.2 involving exon 2 of the *GPC3* gene (arr[hg19] Xq26.2(132996180‐133087198)x1 pat).

The microdeletion was subsequently detected in the 30‐year‐old Caucasian father who had not previously been diagnosed with SGBS. The father exhibited phenotypic characteristics of SGBS including a coarse facial appearance, macrognathia, macrostomia, dental malocclusion, a broad nose, low set ears, rib anomalies, long extremities, and a tall stature. He had been born with a ventricular septal defect and six fingers on each hand. Later, CMA indicated mosaicism for the deletion at Xq26.2 in the paternal grandmother who was phenotypically normal. The mother was phenotypically normal and had a normal female karyotype. A detailed pedigree analysis was obtained and revealed no other affected individuals in the family (data not shown).

The nonconsanguineous parents were interdisciplinary counseled by specialists in fetal medicine and medical geneticists, and the pregnancy was terminated at 15 weeks and 3 days on parental request.

Clinical examination at fetal autopsy revealed numerous abnormalities suggesting the diagnosis of SGBS: postaxial polydactyly of the left hand, broad fingertips, hypoplasia of the right index, preaxial polydactyly of the left foot, craniofacial dysmorphism (turribrachycephaly, bilateral epicanthus, hypertelorism, a broad nasal bridge, anteverted nostrils, low set ears, and macrostomia) (Fig. 1B and C), a lung lobation defect, an annular pancreas, hepatomegaly, and intestinal malrotation. In addition, hypoplasia of skeletal muscles and joint contractures affecting shoulders, hips, and knees was very distinct in the fetus. Muscle biopsies showed severe hypoplasia by standard histopathological approaches, and no further muscle pathology studies were carried out. Estimated fetal weight (55 g) at autopsy was in the normal range for the gestational age.

The X‐chromosome inactivation pattern of the female fetus evaluated on DNA extracted from fetal tissue (HUMARA assay) was normal (44:56). Massively parallel sequencing using NextSeq 500 of the genes *GPC3, OFD1*, and *PIGA* was negative for the presence of disease‐causing variations.

Data on the fetus were submitted to the Decipher database ID 318740.

Procedures were in accordance with the ethical standards of the Danish Ethical committee system. We confirm that that appropriate consent was obtained for presentation of anonymized data.

## Discussion

To our knowledge, this is the first suspected prenatal case of SGBS in a female fetus diagnosed early in pregnancy by CMA.

The published information regarding the presentation of prenatal SGBS is based on findings in second or third trimester pregnancies in male fetuses (Table 1) 6, 7, 8, 9, 10, 11, 12, 13, 14, 15, 16. Macrosomia (11 of 15 cases), polyhydramnios (11 of 15 cases), and kidney and urinary tract anomalies (nine of 15 cases) were the most common findings in the male fetuses with SGBS. These results are in accordance with those found in the study by Cottereau et al., which included a review of pregnancy related data of 36 cases from a larger series of male patients with *GPC3* mutations identified over more than a decade in France 17. In summary, the most frequent prenatal findings associated with SGBS in the French cohort were macrosomia, polyhydramnios, organomegaly, diaphragmatic hernia, cardiac malformations, and hyperechogenic kidneys.

**Table 1:** Comparison of prenatal ultrasound findings in Simpson–Golabi–Behmel syndrome

	Present case	Garavelli et al. 9	Weichert et al. 12	Gertsch et al. 7	Yano et al. 8	Li and McDonald 6	Yamashita et al. 11	Hughes‐Benzie et al. 10	Jedraszak et al. 14	Ochiai et al. 13	Kehrer et al. 15			Magini et al. 16		Total
Sex	Female	Male	Male	Male	Male	Male	Male	Male	Male	Male	Male	Male	Male	Male	Male	
Prenatal findings (wk)																
NT in mm	2.0 (12+5)		5.1 (12)	Increased (12)		3.2 (11+4)								1.3 (na)	3.8 (na)	3/15
Macrosomia			+ (22)	+ (20)	+ (na)	+ (30+5)	+ (29+3)	+ (19)	+ (27)	+ (18)	+ (21+2)	+ (19+2)	+ (17+4)			11/15
Polyhydramnios		+ (na)	+ (22)		+ (na)	+ (30+5)	+ (29+3)	+ (28)	+ (27)		+ (21+2)	+ (19+2)	+ (17+4)		+ (16+3)	11/15
Visceromegaly				+ (20)		+ (30+5)	+ (29+3)		+ (27)							4/15
Congenital diaphragmatic hernia										+ (18)	+ (21+2)	+ (19+2)	+ (17+4)			4/15
Kidney and urinary tract anomalies		+ (na)			+ (na)		+ (29+3)		+ (27)	+ (18)	+ (21+2)		+ (17+4)	+ (20+3)	+ (16+3)	9/15
Craniofacial abnormalities			+ (22)			+ (30+5)						+ (26+0)		+ (20+3)		4/15
Cardiac malformations														+ (20+3)	+ (16+3)	2/15
Mutation	arr Xq26.2(132996180‐133087198)x1 pat	*GPC3,* c.595_597delCGAinsGG	arr Xq26.2(132191191‐133257323)x0 mat	*GPC3,* c.256C>T (p.R86*)	*GPC3*, c.256C>T (p.R86*)	*GPC3*, c.194‐206del113	na	na	*GPC3*, c.662delA	*GPC3*, duplication exon 2b	*GPC3*, c.845dupT	*GPC3*, c.999T>A (p.Y333*)	*GPC3*, duplication exons 3‐7	*GPC3*, c.346G>T (p.E116*)	arr Xq26.2(132834006‐132986815)x0	
Outcome	TOP	CS, 27 wk, liveborn	TOP	CS, 38 wk, liveborn	CS, 37 wk, liveborn	CS, 37 wk, liveborn	CS, 37 wk, liveborn	CS, 34 wk, liveborn	CS, 36 wk, liveborn	TOP	CS, 36+5 wk	Fetal demise	TOP	TOP	TOP	

wk, gestational age in weeks at ultrasonography; NT, nuchal translucency; na, data not available; TOP, termination of pregnancy; CS, cesarean section.

The female fetus in the present case did not express any of the above‐mentioned prenatal clinical features or an increased nuchal translucency, but showed other clear signs of SGBS at fetal autopsy including hand anomalies, craniofacial dysmorphism, hepatomegaly, and intestinal malrotation. Prenatal findings in male fetuses, suggesting an overgrowth syndrome, were all detected later in the second trimester or the third trimester, which may explain why the present female fetus detected early in pregnancy (12+5) did not exhibit any typical SGBS related findings on ultrasound examination.

Yano et al. showed a moderately skewed X‐chromosome inactivation pattern in a carrier female with SGBS, which was proposed as a possible reason for her clinical manifestations 8. In the present report X‐chromosome inactivation analysis performed on DNA extracted from the fetus revealed a normal pattern of X‐chromosome inactivation and could not readily explain the phenotype of the fetus.

The genetic basis of SGBS is not fully understood, and genetic heterogeneity has been proposed as a genetic mechanism. Thus, a possible explanation for the severe phenotype found in our case may alternatively be that the fetus also carried another mutation in either the *GPC3* gene or other SGBS related genes not detected by CMA. Terespolsky et al. reported in 1995 on an infantile lethal variant of SGBS with one patient showing immobile joints of the shoulders and hips prenatally 18 and Fauth et al. recently discovered that a recurrent mutation in the *PIGA* gene leads to SGBS with a severe phenotype [19]. We did not detect disease‐causing variation in the SGBS related genes *GPC3, OFD1*, and *PIGA*. A separate cause of the severe muscle hypoplasia and joint contractures in the fetus could not be excluded, although no history of muscle problems was traced in the family.

## Conclusion

The prenatal diagnosis of SGBS is difficult as the sonographic findings indicating an overgrowth syndrome, such as macrosomia, polyhydramnios, and craniofacial anomalies, are nonspecific and may not appear until late in the pregnancy. In this particular case, CMA was used to establish the likely diagnosis of SGBS in a female fetus with unexpected abnormalities detected in early pregnancy.

## Authorship

HKS: contributed to the conception of the work, the acquisition of data, drafted the work. NB: involved in interpretation of data and drafting the work. VG: involved in the acquisition, analysis, and interpretation of data. MR: involved in the acquisition, analysis, and interpretation of data. IV: contributed to the conception of the work; the acquisition, analysis, and interpretation of data; drafted the work. EMV: involved in substantial contributions to the conception of the work; the acquisition, analysis, and interpretation of data; drafting the work and revising it critically; final approval of the version to be published; accountable for all aspects of the work.

## Conflict of Interest

None declared.
